# The Effects of Various Approaches to Lobectomies on Respiratory Muscle Strength, Diaphragm Thickness, and Exercise Capacity in Lung Cancer

**DOI:** 10.1245/s10434-024-15312-x

**Published:** 2024-04-28

**Authors:** Funda Sirakaya, Ebru Calik Kutukcu, Mehmet Ruhi Onur, Erkan Dikmen, Ulas Kumbasar, Serkan Uysal, Riza Dogan

**Affiliations:** 1 Department of Thoracic Surgery, Faculty of Medicine, Hacettepe University, Ankara, Turkey; 2 Department of Cardiorespiratory Physiotherapy and Rehabilitation, Faculty of Physical Therapy and Rehabilitation, Hacettepe University, Ankara, Turkey; 3 Department of Radiology, Faculty of Medicine, Hacettepe University, Ankara, Turkey

**Keywords:** Video-assisted thoracic surgery, Thoracotomy, Lung cancer, Lobectomy, Respiratory muscles

## Abstract

**Background:**

The most common surgery for non-small cell lung cancer is lobectomy, which can be performed through either thoracotomy or video-assisted thoracic surgery (VATS). Insufficient research has examined respiratory muscle function and exercise capacity in lobectomy performed using conventional thoracotomy (CT), muscle-sparing thoracotomy (MST), or VATS. This study aimed to assess and compare respiratory muscle strength, diaphragm thickness, and exercise capacity in lobectomy using CT, MST, and VATS.

**Methods:**

The primary outcomes were changes in respiratory muscle strength, diaphragm thickness, and exercise capacity. Maximal inspiratory pressure (MIP) and maximal expiratory pressure (MEP) were recorded for respiratory muscle strength. The 6-min walk test (6MWT) was used to assess functional exercise capacity. Diaphragm thickness was measured using B-mode ultrasound.

**Results:**

The study included 42 individuals with lung cancer who underwent lobectomy via CT (*n* = 14), MST (*n* = 14), or VATS (*n* = 14). Assessments were performed on the day before surgery and on postoperative day 20 (range 17–25 days). The decrease in MIP (*p* < 0.001), MEP (*p* = 0.003), 6MWT (*p* < 0.001) values were lower in the VATS group than in the CT group. The decrease in 6MWT distance was lower in the MST group than in the CT group (*p* = 0.012). No significant differences were found among the groups in terms of diaphragmatic muscle thickness (*p* > 0.05).

**Conclusion:**

The VATS technique appears superior to the CT technique in terms of preserving respiratory muscle strength and functional exercise capacity. Thoracic surgeons should refer patients to physiotherapists before lobectomy, especially patients undergoing CT. If lobectomy with VATS will be technically difficult, MST may be an option preferable to CT because of its impact on exercise capacity.

According to data from the World Health Organization International Agency for Research on Cancer 2020, lung cancer is the most common type of cancer in the world. Lung cancer is the leading cause (18.7%) of cancer-related deaths worldwide.^1^ The treatment of early-stage non-small cell lung cancer (NSCLC) is primarily based on surgical resection. The most commonly used surgery for most patients is lobectomy plus systematic lymph node dissection.^2^

Postoperative pulmonary complications (PPC) after thoracotomy have numerous causes, one of which is respiratory muscle dysfunction arising from the alteration of respiratory muscle mechanics and functions due to surgical procedures.^3,4^ Notably, pain is not the only factor contributing to postoperative respiratory muscle dysfunction. Although pain can be reduced with epidural anesthetic, diaphragmatic dysfunction does not show a proportional improvement in response to treatment.^5^ The selection of the surgical incision is thought to be one of the variables contributing to the function of chest wall muscles, which is hypothesized to affect respiratory muscle performance.^6^

Nomori et al.^7^ found no difference in exercise capacity or respiratory muscle strength between limited thoracotomy and video-assisted thoracoscopic surgery (VATS) procedures for patients with lung cancer undergoing lobectomy. In another study, the conventional thoracotomy (CT) group observed a greater decrease in respiratory muscle strength than the muscle-sparing thoracotomy (MST) group in pulmonary resection.^8^ Bernard^6^ found that VATS results in better recovery of respiratory muscle function after lung biopsy than CT. The precise effects and origins of surgery-induced effects on respiratory muscle strength remain unclear. However, no study has examined the effects of all three surgical approaches in lobectomy on respiratory muscle function and exercise capacity.

The diaphragmatic ultrasound parameters measured in healthy individuals are significantly correlated with respiratory function and inspiratory strength.^9^ In a study comparing individuals with chronic obstructive pulmonary disease (COPD) and healthy subjects, the relationship between diaphragm-thinning and the severity of COPD was identified, whereas diaphragm thickness was associated with a decrease in maximal inspiratory pressure %.^10^ In addition to these findings, examination of the literature shows a lack of studies investigating the effects of lobectomy performed with VATS and thoracotomy on diaphragm thickness.

The primary objective of the current study was to assess alterations in respiratory muscle strength, diaphragm thickness, and functional exercise capacity in patients who underwent lobectomy for NSCLC using CT, MST, or VATS. The secondary aim of this study was to compare these alterations in parameters among different surgical methods.

## Materials and Methods

### Patients

This cross-sectional study was conducted at Hacettepe University, Faculty of Medicine, Department of Thoracic Surgery between January and December 2021. The study enrolled patients 18 to 75 years of age who were candidates for lung surgery due to lung cancer. The study excluded individuals who had undergone previous thoracic surgery or neoadjuvant treatment (chemotherapy, radiation therapy) and those with orthopedic or neurologic disorders that could limit mobility. Informed written consent for participation was given by each patient.

This study received ethical approval from the Hacettepe University Clinical Research Ethics Committee (ID:KA-19084). Based on the type of surgery performed, the patients were divided into CT, MST, and VATS groups. The attending surgeon made the decision regarding the surgical strategy based on the location of the tumor.

All the patients were enrolled in a pulmonary rehabilitation program during the postoperative hospitalization period, which included respiratory exercises (thoracic expansion exercises, diaphragmatic breathing, use of incentive spirometry), mobilization, and cough/huffing training.

### Primary Outcomes

The outcomes were assessed on two time frames: the day before surgery and 10 to 15 days after discharge. The primary outcomes were the change in respiratory muscle strength, diaphragm thickness, and exercise capacity from baseline to post-discharge. The secondary outcomes included the incidence of PPC, pulmonary function, dyspnea perception levels, pain perception, and anxiety and depression levels. A qualified physiotherapist performed measurements of respiratory muscle strength, pulmonary function, and exercise capacity, as well as assessment of pain and evaluation of dyspnea. A professional radiologist, who remained blinded to the group allocation to ensure blindness and objectivity, performed diaphragmatic thickness measurements.

The assessment of respiratory muscle was performed according to the guidelines of the American Thoracic Society/European Respiratory Society (ATS/ERS).^11^ A hand-held electronic mouth-pressure device (Micro RPM®;MicroMedical/CareFusion, Kent, UK) was used to measure maximal inspiratory pressure (MIP) and maximal expiratory pressure (MEP).

The 6-min walk test (6MWT) was used to assess functional exercise capacity and was performed in accordance with the ATS recommendations on a 30-m corridor.^12^

The diaphragm thickness was measured using B-mode ultrasonography (Siemens Acuson s3000; Siemens Healthineers, Erlangen, Germany). Measurements were performed using a current with a frequency of 5 to 10 megahertz from the pleural center to the peritoneal center of the diaphragm. Diaphragm thickness was measured while patients were in a semilateral position along the anterior axillary line between the 7th and 9th intercostal spaces. Measurements were taken separately on both sides, at the end of inspiration and expiration, and with a minimum of three repeats to ensure accuracy. To quantify diaphragmatic function DTF, the diaphragmatic thickening fraction (DTF) was calculated using the following formula: (thickness at the end of inspiration-thickness at the end of expiration)/(thickness at the end of expiration) × 100. Values below 20% were considered to be a sign of diaphragm dysfunction.^9^

### Secondary Outcomes

Forced expiratory volume in 1 s (FEV_1_) and forced vital capacity (FVC) were measured using a portable spirometer (Medwelt SP10, Contec Medical Systems Co., Ltd, Hebei, China) according to the ATS/ERS criteria.^13^

Postoperative pain was assessed using the visual analog scale (VAS). The assessment was performed at various time points, including the intensive care unit (ICU) period, before discharge, and during the postoperative follow-up period.^6^

The patients’ degree of dyspnea during daily life was evaluated using the Modified Medical Research Council (mMRC) Dyspnea Scale. Dyspnea assessments were performed twice: first on the day before the surgery and once during the postoperative follow-up period.^14^

The Turkish version of the Hospital Anxiety and Depression Scale (HADS) was used to assess the levels of anxiety and depression.^15^ The assessment was administered while the participants were hospitalized in the ward.

The Melbourne Group Scale (MGS) was used to assess PPC. The presence of four or more of the eight dichotomous parameters listed in the MGS criteria served as the basis for this study’s definition of PPC.^16^ Additionally, the study documented the duration of the participants’ stay in the ICU and the length of their hospitalization.

The Karnofsky Performance Scale (KPS) was used to determine functional impairment levels during the preoperative period. This evaluation established a baseline measurement of the participants’ functional status before their surgical procedures.^17^

### Statistical Analysis

Statistical analyses were performed using the SPSS program (IBM, Ver.25; IBM, New York, NY, USA). The variables were investigated using histograms and the Shapiro–Wilk test. For normal distributions, the paired *t* test was used to compare pre- and post-surgery measurements. In cases of non-normally distributed variables, the Wilcoxon test was used. The McNemar test was used to evaluate the change in pre- and post-surgery nominal variables.

For comparisons among the three different surgical methods (non-normal variables), the Kruskal–Wallis test was used. Post hoc analysis using the Mann–Whitney *U* test was performed when significant differences were observed, with a significance level set at *p* lower than 0.017 to account for the Bonferroni correction. The chi-square test was used to evaluate comparisons among the three groups’ nominal variables. Because pain assessment was performed three times after surgery and did not follow a normal distribution, the Friedman test was used. The significance level for all statistical analyses was set at *p* lower than 0.05.^18^

In the study conducted by Nomori et al.,^4^ G*power analysis (G*Power Software version 3.1.9.3; Heinrich Heine University, Düsseldorf, Germany) was performed using MIP values for pneumonectomy, lobectomy, and segmentectomy both before and 2 weeks after surgery. It was determined that a sample size of 42 individuals would be needed with a power of 90%.

## Results

**Fig. 1:**
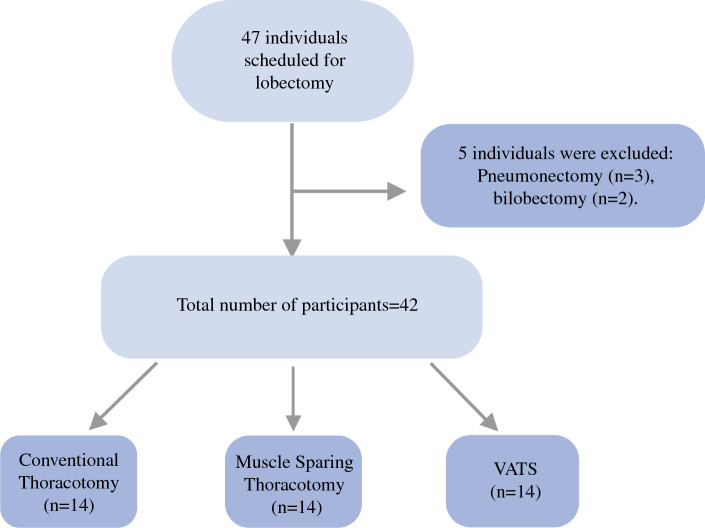
Flowchart

The study included 42 patients who were assigned to one of the three lobectomy procedures (Fig. 1). Assessments were performed on the day before surgery and on postoperative day 20 (range, 17–25 days). The groups were well matched, with no significant differences in age, gender, body mass index (BMI), preoperative spirometric recordings, MIP or MEP values, KPS or mMRC scores, 6MWT distance, or diaphragm thickness (*p* > 0.05; Table 1).

**Table 1:** Baseline characteristics of groups

Veriable	Conventional thoracotomy (*n* = 14)	Muscle-sparing thoracotomy (*n* = 14)	VATS (*n* = 14)	*p* Value
Males: *n* (%)	9 (64.3)	7 (50)	8 (57.1)	0.747
Age (years)	60.36 ± 8.14	60.64 ± 9.24	60.36 ± 9.59	0.995
Height (cm)	165.57 ± 8.09	166 ± 9.49	166.57 ± 8.7	0.955
Weight (kg)	73.93 ± 14.06	69.14 ± 13.86	70 ± 8.41	0.640
BMI (kg/m^2^)	26.91 ± 4.13	26.91 ± 4.129	25.43 ± 4.26	0.387
Smoker	9 (64.3%)	8 (57.1%)	8 (57.1%)	0.217
KPS score (0–100)	95 ± 6.5	95 ± 5.19	97.14 ± 4.69	0.503
mMRC score (0–4)	0.43 ± 0.65	0.43 ± 0.51	0.36 ± 0.5	0.932
FVC (l)	2.72 ± 0.67	2.42 ± 0.70	2.49 ± 0.47	0.379
FEV_1_ (l)	2.39 ± 0.55	2.13 ± 0.62	2.19 ± 0.42	0.374
PEF (l)	6.50 ± 0.98	5.88 ± 1.46	6.04 ± 1.38	0.190
6MWT (m)	510.11 ± 72.48	480.80 ± 52.73	524.01 ± 71.51	0.272
MIP (cmH_2_O)	83.79 ± 18.69	74.93 ± 21.42	76.21 ± 25.03	0.553
MEP (cmH_2_O)	94.36 ± 18.71	91.79 ± 22.95	94.5 ± 21.14	0.740
Surgical-side end of inspiration thickness (cm)	3.07 ± 0.59	3.15 ± 1.02	3.19 ± 0.85	0.984
Surgical-side end of expiration thickness (cm)	2.19 ± 0.52	2.23 ± 0.86	2.24 ± 0.83	0.950
Surgical-side DTF	43.14 ± 25.27	45.00 ± 25.35	49.88 ± 35.75	0.916
Non-surgical-side end of inspiration thickness (cm)	3.19 ± 0.55	3.14 ± 0.69	3.30 ± 0.89	0.773
Non-surgical-side end of expiration thickness (cm)	2.27 ± 0.34	2.29 ± 0.49	2.51 ± 0.73	0.702
Non-surgical-side DTF	41.92 ± 25.19	37.60 ± 13.57	34.03 ± 23.09	0.518

*VATS* video-assisted thoracic surgery, *BMI* body mass index, *KPS* Karnofsky Performance Scale, *mMRC* Modified Medical Research Council, *FVC* forced vital capacity, *FEV*_*1*_ forced expiratory volume in the first second, *PEF* peak expiratory flow, *6MWT* 6-min walk test, *MIP* maximal inspiratory pressure, *cmH*_*2*_*O* centimeter of water, *MEP* maximal expiratory pressure, *DTF* diaphragmatic thickening fraction

**Fig. 2:**
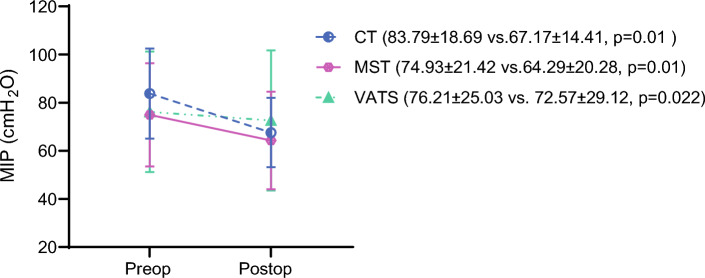
Postoperative change in maximal inspiratory pressure (MIP)

**Fig. 3:**
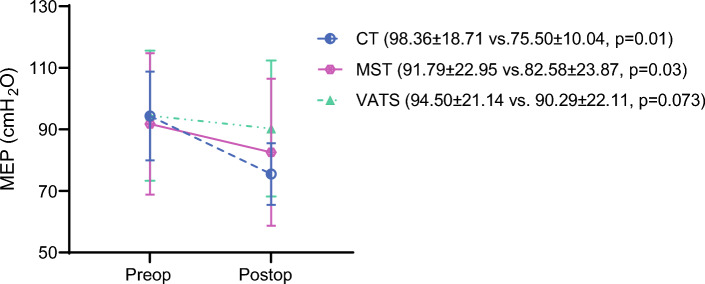
Postoperative change in maximal expiratory pressure (MEP)

**Fig. 4:**
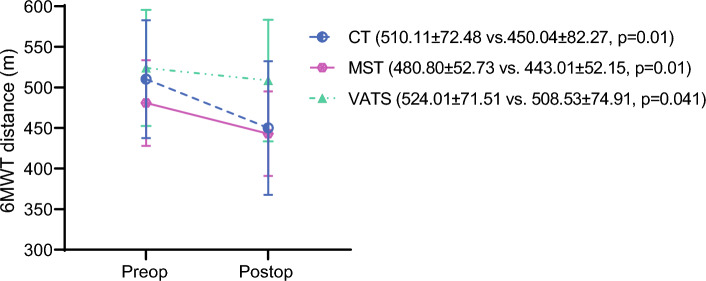
Postoperative change in the 6-min walk test (6MWT)

The average duration of chest tube drainage, the length of ICU stay, and hospitalization were similar between the three procedures (*p* > 0.05; Table 2). According to the MGS scores, the patients in the CT and MST groups had 2% PPC in proportion, and none of the patients in the VATS group had PPC (*p* = 0.331). Pre- and postoperative MIP, MEP, and 6MWT distance changes are shown in Figs. 2, 3 and 4.

**Table 2:** Postoperative changes in respiratory muscle functions, exercise capacity, psychosocial status, dyspnea, and pain perceptions

Veriable	Conventional thoracotomy (*n* = 14)	Muscle-sparing thoracotomy (*n* = 14)	VATS (*n* = 14)	*p* value	Between-groups *p* value
Resected segments	3.64 ± 0.84	3.50 ± 1.02	3.93 ± 0.10	0.475	
Duration of chest tube drainage (days)	5.64 ± 2.76	6.29 ± 4.23	5.07 ± 2.23	0.786	
ICU stay (days)	1.00 ± 0.00	1.00 ± 0.00	1,14 ± 0,36	0.129	
Hospitalization (days)	6.29 ± 2.73	6.79 ± 4.12	6.36 ± 4.89	0.581	
ΔFVC (l)	–0.80 ± 0.33	–0.52 ± 0.33	–0.46 ± 0.28	**0.024**	**0.010**^**a**^
ΔFEV_1_ (l)	–0.72 ± 0.29	–0.47 ± 0.31	–0.41 ± 0.25	**0.017**	**0.009**^**a**^
Δ6MWT (m)	–60.07 ± 26.03	–37.79 ± 24.64	− 15.49 ± 28.62	**< 0.001**	**< 0.001**^**a**^
					**0.012**^**b**^
ΔMIP (cmH_2_O)	–16.21 ± 8.50	–10.64 ± 9.12	–3.64 ± 10.40	**< 0.001**	**< 0.001**^**a**^
ΔMEP (cmH_2_O)	–18.86 ± 14.53	–9.21 ± 6.74	–4.21 ± 10.74	**0.005**	**0.003**^**a**^
HADS score	10.5 ± 4.57	11.42 ± 2.65	5.57 ± 3.5	**0.001**	**0.007**^**c**^
					**< 0.001**^**d**^
Anxiety score	5.93 ± 2.59	6.86 ± 1.75	3.21 ± 1.71	**< 0.001**	**0.004**^**c**^
					**< 0.001**^**d**^
Depression score	4.57 ± 2.34	4.57 ± 1.4	2.36 ± 1.95	**0.006**	**< 0.001**^**d**^
mMRC score (0–4)	1 (0–3)	1 (0–2)	1 (0–2)	0.114	
VAS (cm) ICU	6.85 ± 1.4	6.79 ± 1.4	5.49 ± 1.36	**0.028**	**0.015**^**c**^
VAS (cm) service	4.55 ± 1.7	3.58 ± 1.59	3.66 ± 1.54	0.256	
VAS (cm) after discharge	1.9 ± 1.28	2.3 ± 1.37	1.16 ± 0.67	0.060	

*VATS* video-assisted thoracic surgery, *ICU* ıntensive care unit, *FVC* forced vital capacity, *FEV*_*1*_ forced expiratory volume in the first second, *6MWT* 6-min walk test, *MIP* maximal inspiratory pressure, *cmH*_*2*_*O* centimeter of water, *MEP* maximal expiratory pressure, *HADS* Hospital Anxiety and Depression Scale, *mMRC* Modified Medical Research Council, *VAS* visual analog scale

^a^CT < VATS

^b^CT < MST

^c^VATS < CT

^d^VATS < MST

In the VATS and CT groups, 50% of the patients, and in the MST group, 57.1% of the patients had cardiac comorbidities (*p* = 0.758). In the CT and MST groups, 35.7% of the patients, and in the VATS group, 42.8% of the patients had pulmonary comorbidities (*p* = 0.123).

Postoperative changes in clinical parameters for the three surgical procedures are shown in Table 2. The postoperative changes in FVC, FEV_1_, 6MWT distance, MIP, and MEP values were lower in the VATS group than in the CT group (*p* < 0.017). The 6MWT distance change in the MST group also was lower than in the CT group (*p* < 0.017). The HADS scores were lower in the VATS group than in the CT and MST groups (*p* < 0.017). The VATS group had lower VAS pain scores than the CT group during the ICU period (*p* < 0.017).

Pre- and postoperative differences in diaphragm thickness are shown in Table 3. Only the end of the inspirium diaphragm thickness at the postoperative surgical side was significantly lower than the preoperative thickness (*p* < 0.05). The pre- and postoperative values for other parameters of diaphragm thickness did not differ significantly (*p* > 0.05). The changes in diaphragm thickness were similar between the three procedures (*p* > 0.05).

**Table 3:** Diaphragm thickness changes

Veriable	Preoperative (*n* = 42)	Postoperative (*n* = 42)		P
Surgical-side inspirium (cm)	3.14 ± 0.82	2.80 ± 0.80		**< 0.001**
Surgical-side expirium (cm)	2.22 ± 0.73	2.06 ± 0.74		0.105
Non-surgical-side inspirium (cm)	3.21 ± 0.71	3.39 ± 0.68		0.075
Non-surgical-side expirium (cm)	2.35 ± 0.54	2.51 ± 0.60		0.084
Surgical-side DTF	46.01 ± 28.63	39.98 ± 21.11		0.181
Non-surgical-side DTF	37.85 ± 20.96	37.46 ± 22.38		0.985

	Conventional thoracotomy (*n* = 14)	Muscle sparing thoracotomy (*n* = 14)	VATS (*n* = 14)	
ΔSurgical-side inspirium (cm)	–0.40 ± 0.54	–0.34 ± 0.42	–0.27 ± 0.76	0.616
ΔSurgical-side expirium (cm)	–0.23 ± 0.42	–0.18 ± 0.41	–0.71 ± 0.65	0.260
ΔNon-surgical-side inspirium (cm)	0.25 ± 0.46	0.23 ± 0.46	0.059 ± 0.85	0.767
ΔNon-surgical-side expirium (cm)	0.22 ± 0.41	0.12 ± 0.55	0.12 ± 0.72	0.759
ΔSurgical-side DTF	–4.41 ± 31.91	–6.29 ± 23.22	−7.38 ± 29.63	0.567
ΔNon-surgical-side DTF	–2.24 ± 19.75	5.38 ± 25.46	–4.32 ± 29.49	0.522

*DTF* diaphragmatic thickening fraction

## Discussion

The main finding of our study was that patients with lung cancer who undergo lobectomy with VATS experienced less negative impact on their pulmonary functions, respiratory muscle strength, and exercise capacity than those who undergo CT. Additionally, the patients in the VATS group experienced less depression and anxiety than the patients in the thoracotomy groups. Not only did the MST patients show less impact on exercise capacity than the CT patients, but they also experienced less anxiety and depression. No significant differences were found among the groups in terms of dyspnea and diaphragmatic muscle thickness. During the ICU period, an assessment of pain perception showed that the VATS group experienced significantly less pronounced pain than the CT group.

Upon scrutinizing the research conducted during the early postoperative phase, some experts have observed no notable dissimilarities in the alterations of respiratory muscle strength between the groups that underwent VATS and thoracotomy procedures.^7^ However, other studies have shown that the VATS group had better respiratory muscle strength than the CT group.^19,20^ In a comparison of the MST and CT groups, the MST group generally exhibited better respiratory muscle strength.^8,19^

In our study, when the MIP and MEP values were examined before and after surgery in the VATS group, respiratory muscle strengths were found to be better than in the CT group. These findings align with those of many studies in the literature. The absence of rib retraction and incisions in the latissimus dorsi and serratus anterior muscles in the VATS group likely prevented respiratory muscle dysfunction, explaining why postoperative respiratory muscle strength values in this group were closer to preoperative levels. Respiratory muscle strength measurements are performance-based tests requiring maximum effort. Therefore, better preserved respiratory muscle strengths could have led to better performance in the VATS group with smaller incisions than in the other groups.

According to the literature, patients who undergo VATS tend to have better exercise capacity than those who undergo CT.^21,22^ However, the outcomes are inconsistent for comparison of MST with other groups.^7,23^ Our study also found that VATS may lead to less decline in functional exercise capacity after surgery than CT. In healthy individuals, chest wall compliance affects respiratory muscle strength. Respiratory muscle strength, in turn, is associated with functional capacity.^24^ The CT procedure, which involves incisions in the chest wall, can disrupt chest mobility and thoracic wall compliance. We hypothesize that these disruptions may account for the differences in functional exercise capacity observed among the groups in our study.

Furthermore, the CT group appeared to be more adversely affected in terms of exercise capacity, likely because of respiratory muscle dysfunction. Conversely, both the MST and VATS groups exhibited less dysfunction in the latissimus dorsi and serratus anterior muscles, resulting in no significant difference in exercise capacity changes between these two groups. It is reasonable to assume that the VATS group’s superior preservation of functional exercise capacity after surgery can be attributed to these factors because CT involves a larger incision than VATS, and the location of the incision can cause pain during specific body movements, in addition to potential dysfunction in chest muscles and changes in respiratory mechanics.

Diaphragmatic thickness assessments after thoracic and cardiac surgeries are generally performed during the hospitalization period to determine diaphragmatic dysfunction. The thoracotomy and VATS groups were compared at the postoperative 24th hour in the study conducted by Spadaro et al.,^25^ and it was observed that the VATS group had a lower occurrence of diaphragm dysfunction than the thoracotomy group.

In the pre- and post-surgery analyses, a statistically significant difference was detected only in the thickness of the diaphragm post-inspiration on the side that had surgery when the data were analyzed without any grouping. This difference is believed to be due to parenchymal loss on the surgical side. Because of the reduced load on the diaphragm on the surgical side, a decrease in the cross-sectional area occurred during the postoperative period. A decrease in diaphragm thickness has been associated with the severity of chronic diseases such as COPD and a reduction in inspiratory muscle strength.^10^ In our study, the postoperative decrease in muscle thickness could be considered as an important assessment for health care professionals working in this field.

The findings of our study are consistent with those of earlier studies in this field, as documented in those studies,^26–28^ which also did not identify significant differences between the hospital length of stay and the incidence of postoperative complications when VATS was compared with thoracotomy procedures. Our research showed that no significant variations were observed between the groups concerning postoperative pulmonary complications, use of chest tubes, ICU length of stay, and hospitalization. It is worth mentioning that our medical center frequently discharges patients either on the same day or the following day after removal of the chest tube, based on the presence or absence of any other complications. The similar duration of chest tube use may be a factor in the comparable hospital lengths of stay observed across the groups. Our study found that the three surgical techniques had similar success rates.

Studies examining early postoperative pain have shown that patients who undergo VATS resection experience lower levels of pain than those who undergo thoracotomy.^27,29,30^ However, in another study, pain levels remained similar in the thoracotomy and VATS groups, even when measured up to 12 months after surgery.^31^

Pain scores were evaluated between the MST and CT groups in a meta-analysis that included 1083 patients. According to the findings, only the pain scores on the seventh day were significantly lower in the muscle-sparing thoracotomy group, with similar scores on the first day in 30 days.^28^

In another study comparing MST and CT, pain scores remained similar during hospitalization and up to 48 weeks after discharge.^32^ In our study, it was observed that the postoperative ICU pain scores of the VATS group were significantly lower than those of the CT group. However, pain scores did not differ significantly between the groups in the postoperative ward or during the post-discharge follow-up period. These results are consistent with those of many studies in the literature.^28,31,32^ Nevertheless, it is evident that there is still ambiguity and contradicting evidence in the literature about pain. One possible explanation for the lower pain scores in the VATS group, particularly in the ICU, could be attributable to the surgical approach itself. In VATS procedures, there is less rib mobility, potentially resulting in less damage to the intercostal nerves. In addition, the absence of rib retraction and the avoidance of incisions in the latissimus dorsi and serratus anterior muscles in VATS may contribute to reduced pain development. However, it is essential to acknowledge that pain perception can vary widely among individuals, and the experience of pain is influenced by numerous factors beyond the surgical technique alone. Therefore, the management of postoperative pain should be individualized, considering patient-specific factors and preferences.

Studies on pulmonary functions in lung resection surgeries generally either report no significant differences between surgical techniques or demonstrate that VATS procedures result in less harmful impacts on FEV_1_ and FVC values than CT.^7,28–30,33,34^ These findings align with the results of our study. We observed that the VATS group experienced fewer adverse effects on pulmonary functions after surgery than the CT group. Nagahiro et al.^30^ suggested that the preserved postoperative pulmonary functions observed during the first week in patients who underwent VATS lobectomy could be attributed to reduced pain levels. However, they found no disparities in pain scores between the two groups in the second week. They attributed this difference in pulmonary function outcomes to the preservation of respiratory muscle functions, particularly the latissimus dorsi and serratus anterior muscles in VATS lobectomy cases.

Although pain undoubtedly affects postoperative pulmonary function parameters, it is important to recognize that pain is not the sole determinant of these changes. In our study, we did not observe significant differences in pain assessments performed during the same time frame as the pulmonary function tests. The existence of muscle incisions in typical thoracotomy procedures is a potential explanation for the differences in postoperative pulmonary function alterations across the groups. These muscle incisions can disrupt the mechanics of the respiratory muscles and thoracic wall compliance. This disruption could elucidate why the CT group exhibited more pronounced adverse effects on pulmonary functions after surgery than the VATS group, in which such muscle incisions were notably absent. In summary, although pain is undoubtedly a contributing factor to postoperative pulmonary function, it is essential to consider a multifaceted interplay of factors, including surgical technique and the presence or absence of muscle incisions, when assessing and interpreting postoperative respiratory outcomes.

Anxiety, depression, and stress are common issues during the perioperative period of thoracic surgery. In addition, these emotional problems have physiologic effects.^35^ Insufficient research has been conducted to examine the development of anxiety and depression in patients who underwent lobectomy through thoracotomy and VATS. Hopkins et al.^36^ compared the anxiety and depression scores of patients undergoing lung resection with VATS and thoracotomy and did not find significant difference. Another study by Park et al.^37^ noted that thoracotomy posed a greater risk factor for depression and anxiety than minimally invasive methods. Our research indicated that the VATS group exhibied lower HADS scores than the CT and MST groups. Therefore, the VATS group had a lower risk for the development of depression and anxiety. The psychosocial status of a patient is thought to be profoundly influenced by the selection of surgical incision technique. As the incision size increases, the pain experienced and patient mobility limitations may contribute to anxiety and depression. The outcomes obtained in this study are considered to be an anticipated result because the initial inquiry of the research participants after the procedure was whether their surgery was open or closed.

The primary limitation of this study was the inability to randomize and blind the surgical procedure because the surgical decision was the basis for the method used. Blinding could not be performed for the physiotherapy assessments. We also assessed exercise capacity using the 6MWT, a submaximal test, instead of the CPET, the maximal test.

The strength of our study was being the first to evaluate diaphragm muscle thickness in patients who had undergone lobectomy. Diaphragm thickness measurements were performed blinded. Our study was the first to simultaneously assess respiratory muscle strength and exercise capacity in individuals undergoing lobectomy using VATS, CT, or MST.

In conclusion, lobectomy negatively affects respiratory muscle strength, diaphragm thickness, and functional exercise capacity. The impact of VATS and MST surgical techniques on these parameters is similar, but VATS is better than CT. The MST group showed less impact on exercise capacity than the CT group. However, because all the groups experienced negative effects on respiratory muscle strength, functional exercise capacity, and diaphragm thickness compared with the preoperative period, it is essential to implement a respiratory muscle training and exercise program that starts before surgery and continues through the postoperative period to minimize the adverse effects of surgery. Therefore, thoracic surgeons should refer patients to physiotherapists before lobectomy, especially patients undergoing CT, for preoperative preparation, for patient education, and to minimize the decrease in respiratory muscle functions and exercise capacity. Studies are needed to investigate the effectiveness of pre- and postoperative rehabilitation approaches among VATS, CT, and MST patients. If lobectomy with VATS will be technically difficult, MST may be a preferable option compared with CT because of its impact on exercise capacity.
